# Temporal Trends in the Impact of Preterm Birth on Low Birthweight Rates in Greece

**DOI:** 10.7759/cureus.105303

**Published:** 2026-03-16

**Authors:** Nikolaos Vlachadis, Nikolaos Machairiotis, Dimos Sioutis, Konstantinos Louis, Charalampos Theofanakis, Chrissi Christodoulaki, Periklis Panagopoulos

**Affiliations:** 1 Third Department of Obstetrics and Gynecology, National and Kapodistrian University of Athens, Medical School, Attiko University Hospital, Athens, GRC; 2 Department of Obstetrics and Gynecology, General Hospital of Chania "O Agios Georgios", Chania, GRC

**Keywords:** greece, low birthweight, premature labour, preterm births, time trends

## Abstract

Background and objectives: In Greece, both the preterm birth rate (PBR) and the low birthweight rate (LBWR) have risen sharply in recent decades, reaching among the highest levels in high-income countries and posing a substantial public health concern. This study aimed to quantify the contribution of preterm births to the national LBW burden and to determine the extent to which the rise in PBR has driven the increase in LBWR.

Methods: The analysis included 4,585,090 live births recorded in Greece from 1980 to 2023, stratified by gestational age and birthweight. Temporal trends were assessed using joinpoint regression analysis. For each segment between two joinpoints, the annual percent change (APC) was calculated with 95% confidence intervals (CIs), and statistical significance was set at p < 0.05.

Results: From 1980 to 2023, both LBWR and PBR increased substantially and were strongly correlated (rho = 0.858, 95% CI: 0.725-0.929, p < 0.001). The preterm LBWR remained stable from 1987 to 2004, then rose significantly thereafter (APC = 0.5, 95% CI: 0.2-0.9, p = 0.027). In contrast, the term LBWR increased during the 1980s and 1990s, declined significantly from 2002 to 2017 (APC = -3.5, 95% CI: -6.2 to -2.7, p = 0.033), and then stabilized. The population attributable risk (PAR, %) of LBW due to prematurity increased from a low of 25.8% in 1991 to a historic peak of 68.2% in 2021 (67.1% in 2023), with statistically significant rises during 1991-2004 (APC = 2.5, 95% CI: 1.3-7.7, p = 0.024), 2004-2009 (APC = 9.1, 95% CI: 2.4-13.9, p = 0.021), and 2009-2023 (APC = 1.4, 95% CI: 0.7-2.0, p = 0.014). The proportion of LBW neonates born preterm rose from a minimum of 27.8% in 1991 to a peak of 72.0% in 2021 (71.2% in 2023). The entire increase in LBWR between 1991 and 2023 was fully explained by the concurrent rise in PBR.

Conclusions: This nationwide study demonstrates that preterm birth is the principal driver of Greece’s high LBWR. The population risk of LBW attributable to prematurity has increased markedly over the past three decades, and the sustained rise in LBWR is entirely accounted for by the parallel increase in PBR. These findings underscore the urgent need for targeted, evidence-based clinical and public health strategies to reduce prematurity and improve perinatal outcomes.

## Introduction

Preterm birth and low birth weight (LBW) remain two of the most critical perinatal challenges and pressing global public health concerns [1-3]. Preterm birth (<37 completed weeks of gestation) is the leading cause of neonatal mortality and is linked to a broad range of adverse outcomes, including impaired growth, developmental and cognitive deficits, early onset of chronic diseases, and long-term physical, neurodevelopmental, and socioeconomic consequences. In 2020, more than 13 million infants - approximately 10% of all live births - were born preterm worldwide, with no measurable decline over the preceding decade [2].

LBW (<2500 g) is a major determinant of neonatal morbidity and mortality and a key predictor of health outcomes across the lifespan [4-6]. It is associated with early adverse outcomes, such as elevated risk of neonatal mortality as well as long-term consequences and an increased risk of chronic conditions, including metabolic syndrome and cardiovascular disease. In 2020, an estimated 20 million newborns - about 15% of all live births - were classified as LBW, showing only minimal improvement compared with 2000. Nearly three-quarters of all LBW births, as well as almost two-thirds of preterm births, occur in Southern Asia and sub-Saharan Africa [2,4].

LBW may result from preterm birth, being small for gestational age (SGA), or a combination of both [4]. SGA refers to newborns whose birth weight is below the 10th percentile for their gestational age and sex. These neonates generally correspond to cases of fetal growth restriction (FGR), defined as an estimated fetal weight below the 10th percentile for gestational age and sex [7]. The proportionate contribution of preterm versus term LBW infants is influenced by the level of a country’s economic development; in resource-limited settings, term LBW births frequently constitute the predominant component of the total LBW burden [8].

Globally, preterm birth accounts for more than 70% of neonatal deaths, while over 80% of all neonates who die are born with LBW [3]. LBW serves as an important - though imperfect - proxy for prematurity when accurate gestational age is unavailable. The increasing prevalence of LBW infants, despite advances in infant survival, is likely to impose a substantial future burden on both health systems and socioeconomic resources [6]. Traditionally, preterm birth and LBW have been treated as separate conditions, although they frequently overlap. Evaluating them in isolation underestimates the spectrum of risks faced by small newborns. Infants who are both preterm and LBW represent the most vulnerable group, with the highest risk of severe complications, need for intensive care, or death [3,9].

In Greece, both the preterm birth rate (PBR) and the low birthweight rate (LBWR) have risen sharply since the early 1990s, reaching among the highest levels in high-income countries and representing a substantial public health concern [10,11]. Given the well-established relationship between preterm birth and LBW, this study has two aims: first, to quantitatively assess the contribution of preterm births to the total LBW burden in Greece; and second, to examine the association between trends in the PBR and changes in the LBWR in the country.

## Materials and methods

Study population

This study utilized publicly accessible data from the Hellenic Statistical Authority [12], derived from official birth certificate records. The dataset encompassed all live births registered in Greece from 1980 to 2023, stratified by gestational age at birth (measured in completed weeks) and birthweight (measured in grams).

Inclusion and exclusion criteria

During the 44-year study period (1980-2023), a total of 4,605,769 live births were recorded in Greece. Of these, 20,679 cases (0.45%) were excluded due to missing data on gestational age and/or birthweight. The remaining 4,585,090 live births (99.55%) with complete data on both gestational age and birthweight were included in the final analysis.

Study parameters

The PBR was first calculated for each year from 1980 to 2023, expressed as the number of preterm live births per 100 total live births (%). The overall annual LBWR was then determined, also expressed per 100 total live births (%). Annual LBWR were also calculated separately for preterm births (preterm low birthweight rate, PLBWR), expressed as the number of preterm LBW newborns per 100 preterm live births (%), and for term births, defined as those occurring at ≥37 gestational weeks (term low birthweight rate, TLBWR), expressed as the number of term LBW neonates per 100 term live births (%).

For each year, the relative risk (RR) of LBW for preterm neonates compared with those who completed at least 37 weeks of gestation was calculated as the ratio PLBWR ÷ TLBWR, representing the incidence ratio of LBW in preterm vs. term live births. Using the RR together with the PBR, which reflects the prevalence of preterm births in the population, the population attributable risk (PAR, %) of preterm birth for LBW was calculated, following previously described methods [13].

Statistical analysis

Data analysis was conducted using Microsoft Excel 2010 (Microsoft Corporation, Redmond, WA, USA). Temporal trends were assessed with the Joinpoint Regression Program, version 5.2.0 (National Cancer Institute, Bethesda, USA), which identifies joinpoints marking periods of statistically significant changes in trend. For each segment between two joinpoints, the annual percent change (APC) was calculated, with a maximum of seven segments allowed. Results are presented with 95% confidence intervals (CIs), and statistical significance was set at p < 0.05.

To estimate the contribution of preterm births to the change in the LBWR between Year A and Year B, we used the PAR (%) for each year. The PAR (%) represents the proportion of LBW cases attributable to preterm birth. For each year, the preterm-attributable LBWR was calculated by multiplying the overall LBWR by the corresponding PAR (%). The absolute changes in the overall LBWR and in the preterm-attributable LBWR between the two years were then computed. The contribution (%) of the PBR to the increase in LBWR was estimated as the ratio of the change in the preterm-attributable LBWR to the change in the overall LBWR, multiplied by 100. This metric indicates the proportion of the observed increase in the LBWR that is attributable to changes in the prevalence and impact of preterm birth.

## Results

**Figure 1:**
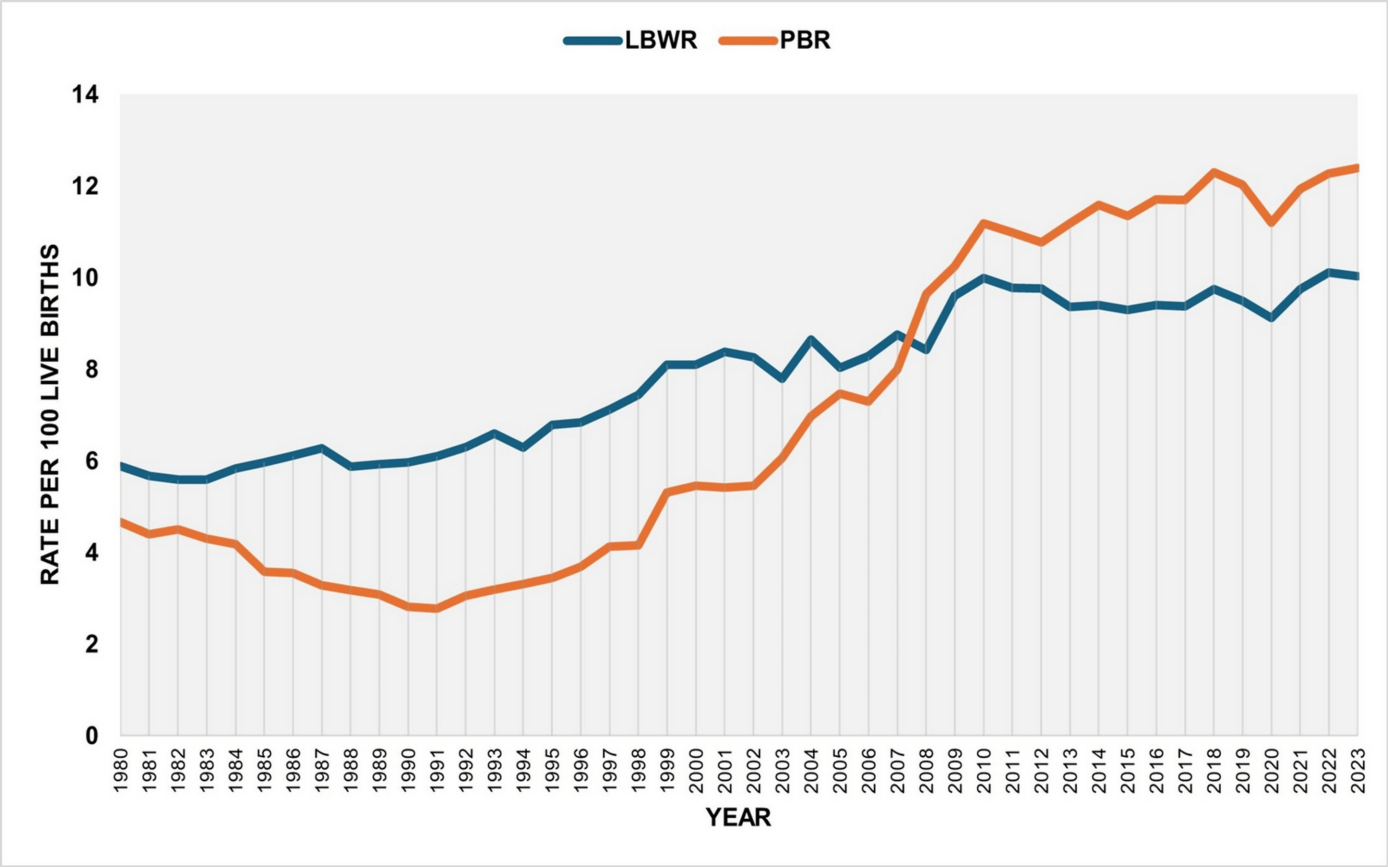
Low birthweight rate (LBWR) and preterm birth rate (PBR) (per 100 live births) in Greece, 1980-2023

During 1980-2023, both the LBWR and the PBR increased substantially. The time trends of LBWR and PBR in Greece over this period have been described in detail elsewhere [10,11]. The PBR rose more rapidly and surpassed the LBWR in 2008, after which the annual number of preterm births per 100 live births consistently exceeded that of LBW neonates. The LBWR and the PBR showed a strong correlation over the entire period from 1980 to 2023 (rho = 0.858, 95% CI: 0.725 to 0.929, p < 0.001), which was even stronger during the period of rapid increase from 1991 to 2010 (rho = 0.921, 95% CI: 0.755 to 0.976, p < 0.001) (Figure 1).

**Figure 2:**
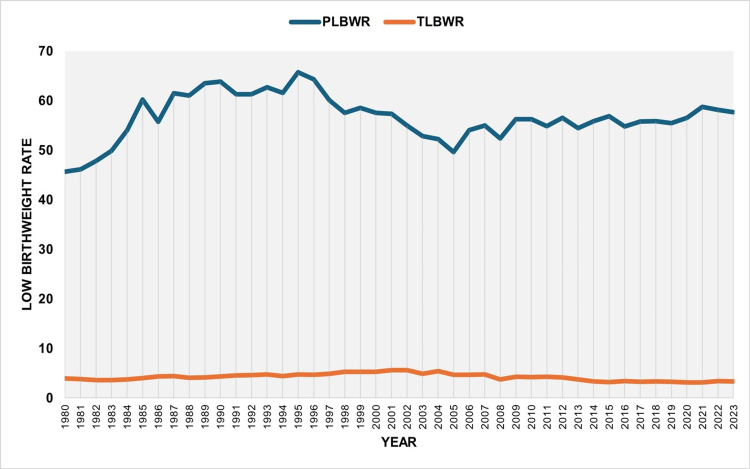
Preterm low birthweight rate (PLBWR) (per 100 preterm live births) and term low birthweight rate (TLBWR) (per 100 term live births) in Greece, 1980-2023

**Figure 3:**
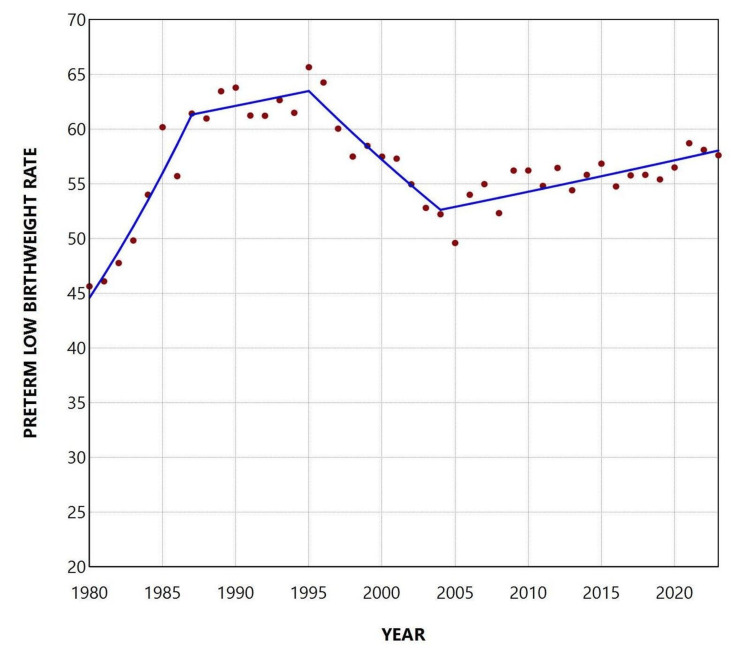
Trends in the preterm low birthweight rate (per 100 preterm live births) in Greece, 1980-2023

**Figure 4:**
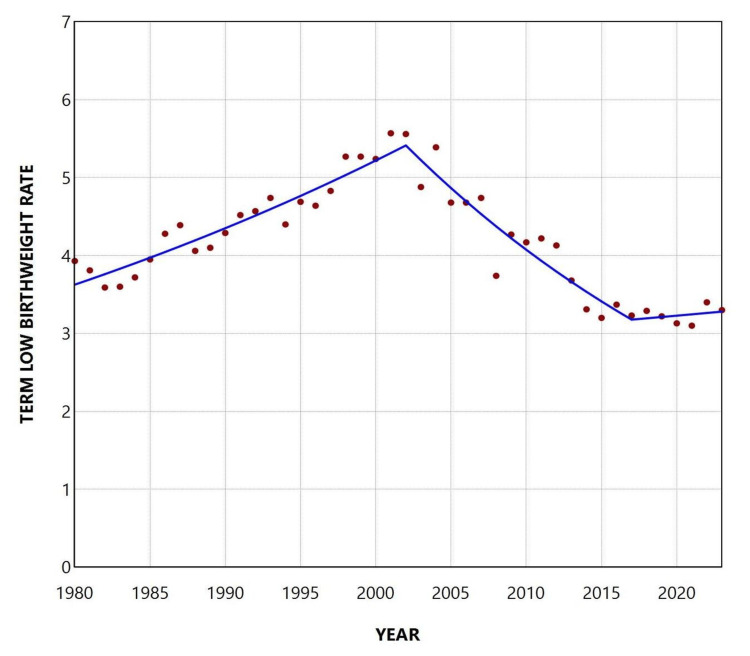
Trends in the term low birthweight rate (per 100 term live births) in Greece, 1980-2023

The total PLBWR was 55.7% over the entire period, ranging from a minimum of 45.64% in 1980 to a maximum of 65.67% in 1995. The trend increased between 1980 and 1987 (APC = 4.7, 95% CI: 3.6 to 6.6, p < 0.001), remained statistically unchanged from 1987 to 2004, and showed a renewed upward trend during the more recent period from 2004 to 2023 (APC = 0.5, 95% CI: 0.2 to 0.9, p = 0.027). The overall TLBWR was 4.2% from 1980 to 2023, with a peak of 5.57% in 2001 and a low of 3.10% in 2021. An upward trend was observed during the 1980s and 1990s (1980-2002: APC = 1.8, 95% CI: 1.4 to 2.3, p = 0.006), followed by a significant decline from 2002 to 2017 (APC = -3.5, 95% CI: -6.2 to -2.7, p = 0.033), and stabilization thereafter (Figures 2-4 and Tables 1, 2).

**Table 1:** Trends in the preterm low birthweight rate in Greece, 1980-2023 Trends were calculated using joinpoint regression analysis. * indicates a statistically significant P-value.

Segment	Annual percent change	95% confidence interval	P-value
1980-1987	4.7	3.6 to 6.6	<0.001*
1987-1995	0.4	-1.1 to 2.0	0.393
1995-2004	-2.1	-5.1 to 0.2	0.054
2004-2023	0.5	0.2 to 0.9	0.027*

**Table 2:** Trends in the term low birthweight rate in Greece, 1980-2023 Trends were calculated using joinpoint regression analysis. * indicates a statistically significant P-value.

Segment	Annual percent change	95% confidence interval	P-value
1980-2002	1.8	1.4 to 2.3	0.006*
2002-2017	-3.5	-6.2 to -2.7	0.033*
2017-2023	0.5	-2.2 to 7.8	0.602

**Figure 5:**
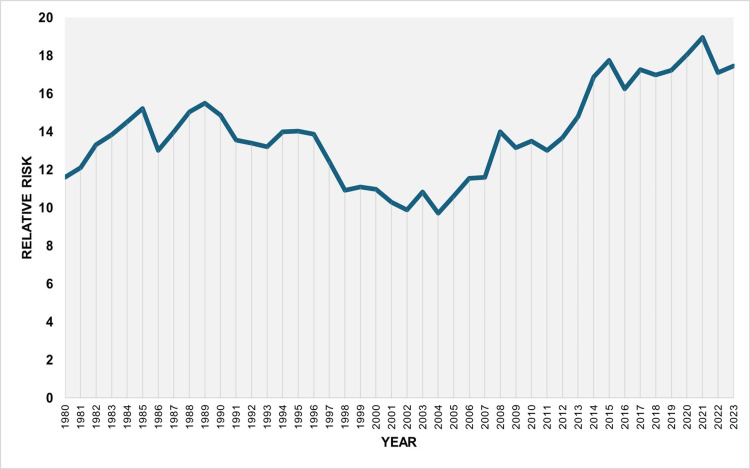
Relative risk of low birthweight among preterm compared to term neonates in Greece, 1980-2023

**Figure 6:**
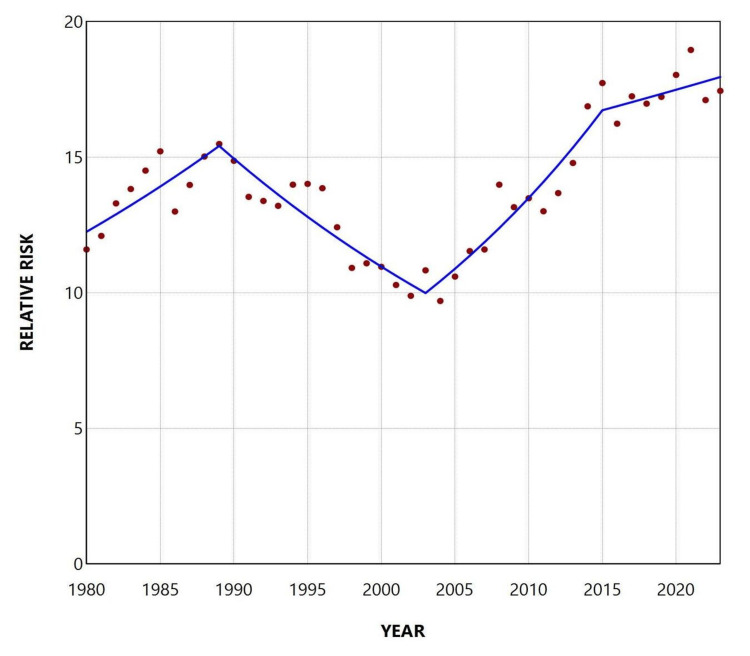
Trends in the relative risk of low birthweight among preterm compared to term neonates in Greece, 1980-2023

The RR of LBW among preterm neonates compared to those born ≥ 37 weeks of gestation was 13.2 overall, with an all-time low of 9.7 in 2004 and a historic maximum of 19.0 in 2021. An increasing trend was observed from 1980 to 1989 (APC = 2.6; 95% CI: 0.9 to 5.0; p = 0.022), followed by a declining trend between 1989 and 2003 (APC = -3.1; 95% CI: -4.6 to -2.0; p = 0.032). Since 2003, the trend has shown upward fluctuations without statistical significance (Figures 5, 6, and Table 3).

**Table 3:** Trends in the relative risk of low birthweight among preterm compared to term neonates in Greece, 1980-2023 Trends were calculated using joinpoint regression analysis. * indicates a statistically significant P-value.

Segment	Annual percent change	95% confidence interval	P-value
1980-1989	2.6	0.9 to 5.0	0.022*
1989-2003	-3.1	-4.6 to -2.0	0.032*
2003-2015	4.4	-1.0 to 9.1	0.060
2015-2023	0.9	-3.8 to 2.9	0.465

**Figure 7:**
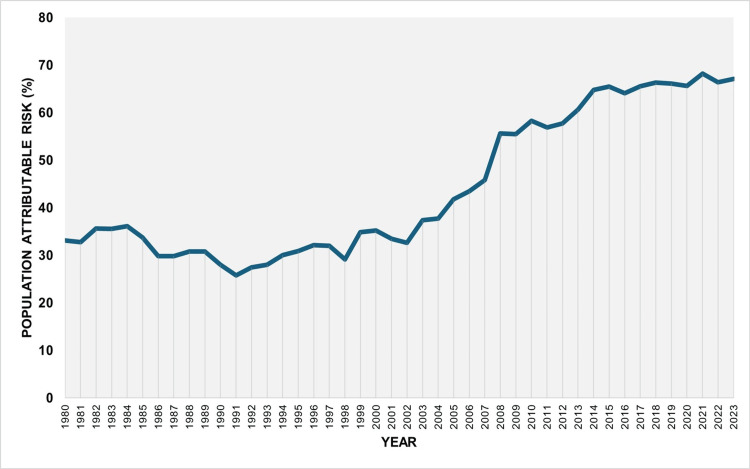
Population attributable risk (%) of low birthweight due to preterm births in Greece, 1980-2023

**Figure 8:**
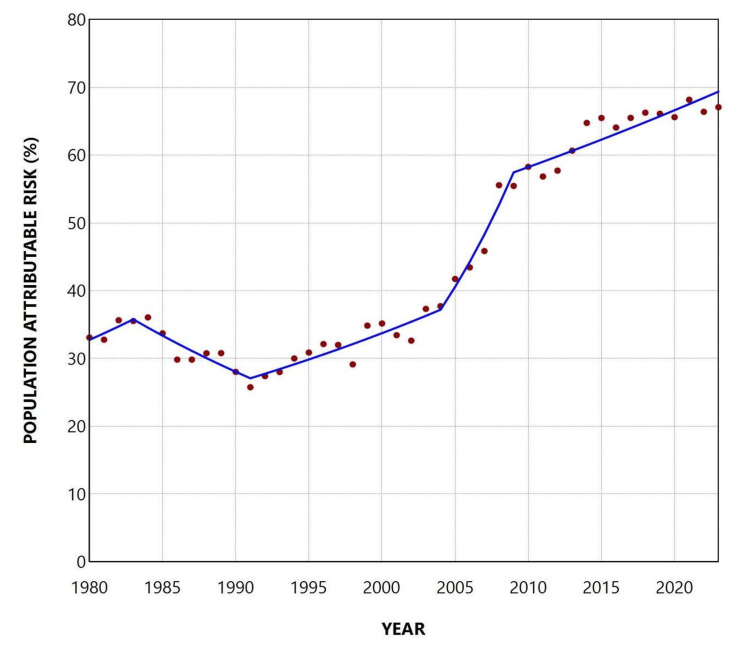
Trends in the population attributable risk (%) of low birthweight due to preterm birth in Greece, 1980-2023

The PAR (%) for the entire period 1980-2023 was 45.1%. The trend was not statistically significant until 1991, when the rate reached its lowest recorded value of 25.8%. However, from 1991 onward, the trend was consistently increasing, with an APC of 2.5 (95% CI: 1.3 to 7.7, p = 0.024) during 1991-2004, a steeper increase during 2004-2009 (APC = 9.1; 95% CI: 2.4 to 13.9; p = 0.021), and a slower but still significant rise from 2009 onward (APC = 1.4; 95% CI: 0.7 to 2.0; p = 0.014). The PAR (%) reached its historic maximum of 68.2% in 2021, declined slightly to 67.1% in 2023, and has remained consistently above 65% since 2017 (Figures 7, 8, and Table 4).

**Table 4:** Trends in the population attributable risk (%) of low birthweight due to preterm births in Greece, 1980-2023 Trends were calculated using joinpoint regression analysis. * indicates a statistically significant P-value.

Segment	Annual percent change	95% confidence interval	P-value
1980-1983	3.0	-2.1 to 10.2	0.231
1983-1991	-3.4	-7.1 to 2.7	0.108
1991-2004	2.5	1.3 to 7.7	0.024*
2004-2009	9.1	2.4 to 13.9	0.021*
2009-2023	1.4	0.7 to 2.0	0.014*

**Figure 9:**
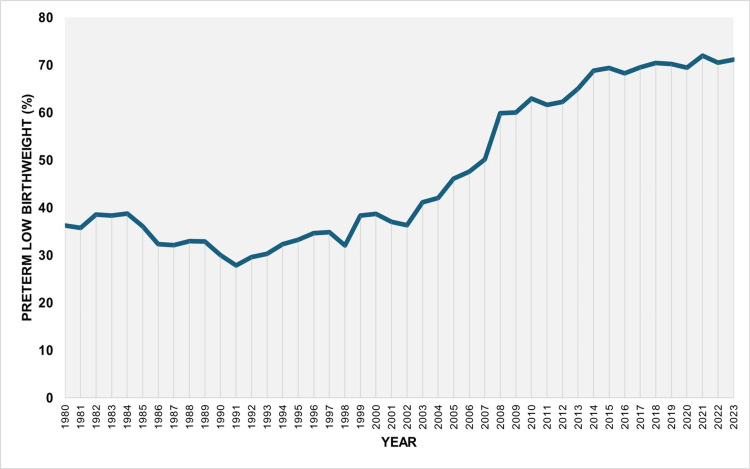
Proportion of preterm births among low birthweight births in Greece, 1980-2023

**Figure 10:**
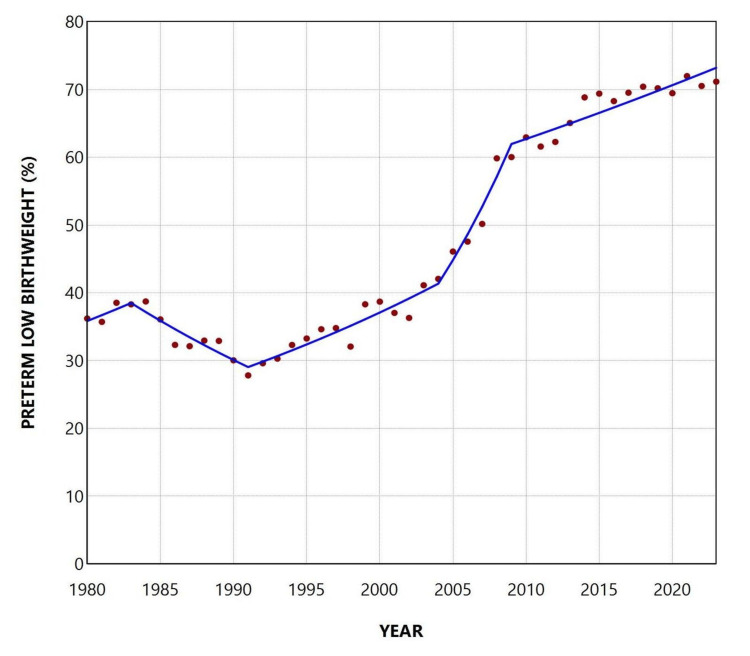
Trends in the proportion of preterm births among low birthweight births in Greece, 1980-2023

Over the entire period from 1980 to 2023, 48.7% of LBW neonates were born preterm. Following an initial period of stability, the proportion of preterm LBW newborns among all LBW births increased markedly, from an all-time low of 27.8% in 1991 to a historic peak of 72.0% in 2021, with a slight decrease to 71.2% in 2023. In each of the past 10 years (since 2014), preterm neonates have consistently accounted for more than 67% of all LBW newborns in Greece (Figures 9, 10, and Table 5).

**Table 5:** Trends in the proportion of preterm births among low birthweight births in Greece, 1980-2023 Trends were calculated using joinpoint regression analysis. * indicates a statistically significant P-value.

Segment	Annual percent change	95% confidence interval	P-value
1980-1983	2.4	-2.3 to 9.1	0.297
1983-1991	-3.5	-6.9 to 3.0	0.117
1991-2004	2.8	1.5 to 7.5	0.022*
2004-2009	8.4	1.7 to 12.7	0.022*
2009-2023	1.2	0.6 to 1.8	0.016*

Between 1991 and 2023, the LBWR increased by 3.94 percentage points, from 6.09% to 10.03%. The portion of the LBWR attributable to preterm births rose from 1.57% (calculated as 0.2576 × 6.09) in 1991 to 6.73% (0.6708 × 10.03) in 2023, reflecting an absolute increase of 5.16 percentage points. This indicates that the rise in preterm births accounted for approximately 131% (5.16/3.904) of the observed increase in LBWR during that period. A contribution exceeding 100% suggests that the entire rise in LBWR since 1991 was more than fully accounted for by the increase in PBR.

## Discussion

In the present study, analysis of official national data highlighted the substantial contribution of preterm births to the overall incidence of LBW in Greece. Furthermore, it was found that the marked increase in the LBWR in the country during the period 1991-2023 is entirely attributable to the simultaneous rise in preterm births.

During the study period, and particularly since 1991, Greece experienced a dramatic upward trend in both the PBR and the LBWR. The present analysis revealed that the temporal trajectories of these two indicators were closely correlated, a relationship that was especially pronounced during the two decades of the steepest increase - the 1990s and 2000s. Since 2010, both indicators have continued to rise, albeit at a slower pace, resulting in Greece ranking among the countries with the highest rates of preterm births and LBW infants among all developed nations [10,11].

Because a shortened gestational duration is by far the major risk factor for LBW, we sought to quantitatively assess the contribution of preterm births to LBW incidence by calculating LBWR separately for preterm and term births. Typically, the PLBWR reflects the proportion of LBW births attributable to prematurity, whereas the TLBWR reflects the proportion attributable to SGA. For PLBWR, after a period of stabilization between 1987 and 2004, an upward trend was observed over the past two decades. In contrast, TLBWR showed a marked decline during the same period, suggesting a relative reduction in the incidence of SGA or growth restriction in Greece. This may indicate improvements in antenatal care for pregnant women in recent years [8]. However, the concurrent rise in PLBWR implies that such improvements may have had a greater impact on fetal growth in term pregnancies than on the prevention of preterm deliveries, highlighting the persistent challenge of reducing prematurity rates.

Overall, the PLBWR was 13.2 times higher than the TLBWR (55.7% vs. 4.2%). The RR of LBW for preterm compared with term infants fluctuated considerably, from 9.7 in 2004 to 19.0 in 2021, and finally to 17.5 in 2023. The PAF (%) for preterm birth increased markedly from 25.8% in 1991 to 68.2% in 2021 and remained consistently above 65% since 2017. This trend reflects the concurrent rise in both the PLBWR and the RR of LBW in preterm compared with term neonates. The proportion of LBW infants who were born preterm, although very low at 27.8% in 1991, rose to 72.0% in 2021 and stayed above 67% throughout the past decade. Using these data, it was possible to estimate that the rise in LBWR since 1991 was entirely attributable to the increase in the PBR in the country. Specifically, the contribution of preterm births to the increase in total LBWR from 1991 to 2023 was estimated at 131%. This value, exceeding 100%, indicates that the entire increase in LBWR was more than fully explained by the rise in preterm births, which is accounted for by the concurrent decline in the proportion of LBW among term births over the same period. In other words, the interpretation of this finding is that if the contribution of preterm births to LBW births in Greece had remained unchanged between 1991 and 2023, not only would the large increase in LBWR disappear, but the total LBWR in 2023 would have been lower than the LBWR observed in 1991.

In 2023, the PLBWR among preterm neonates was 57.62%, 17.5 times higher than the TLBWR (3.30%). The PAF (%) of LBW births due to preterm delivery was 67.1%, indicating that roughly two-thirds of LBW births in Greece could have been avoided if all deliveries had occurred at term. Overall, 71.2% of LBW neonates were born preterm, underscoring the substantial contribution of prematurity to the national LBW burden. By comparison, an analysis of 2014-2018 birth data from Asian and African countries with high PBRs reported an overall LBWR of 13.6%, of which only about 40% were preterm, with only Pakistan marginally exceeding 50% [3].

The increase in the LBWR in Greece from 1991 to 2023 could have resulted from an increase in the contribution of preterm births, term births, or both. Our data showed that the increase resulted entirely from the rise in the contribution of preterm births, because in 2023, compared with 1991, the proportion of term births among total births decreased (from 97.23% in 1991 to 87.61% in 2023), and the proportion of LBW births among term births (TLBWR) also decreased (from 4.52% in 1991 to 3.30% in 2023). Furthermore, the increased contribution of preterm births to the total LBWR could be the result of an increased proportion of preterm births among total births (PBR), an increased proportion of LBW births among preterm births (PLBWR), or both. Our data showed that the increase in LBWR from 1991 to 2023 is attributable only to the increase in the PBR (from 2.77% in 1991 to 12.39% in 2023), since the PLBWR in 2023 was lower than that in 1991 (57.62% vs 61.25%, respectively).

In Greece, perinatal indicators have deteriorated sharply since the early 1990s. At least two major factors may have contributed to this trend. This period was marked by rapid changes in the reproductive behavior of Greek women, leading to a steep rise in the age at childbearing and a substantial increase in the proportion of births among older mothers [14]. Advanced maternal age is a well-established risk factor for adverse perinatal outcomes and is associated with an increased incidence of both preterm delivery and the birth of LBW infants [15,16]. A second factor, closely related to the first, is the pronounced upward trend in births from multiple pregnancies observed in Greece during this time [17]. Multiple pregnancies result in a much higher proportion of both preterm births and LBW neonates compared with singleton pregnancies [13,18,19]. The adoption of best clinical practices regarding the number of embryos transferred could significantly reduce the rate of multiple gestations following assisted reproductive technology (ART) treatment. Nevertheless, the absolute number of multiple pregnancies is expected to rise further as the use of ART to address infertility increases. Our findings indicate that, since the early 1990s, trends in PBR, LBWR, and multiple birth rate have followed a parallel upward trajectory. One report has described the close link between the rise in multiple pregnancies and the increase in the PBR in Greece, estimating that this factor accounts for more than 80% of the observed upward trend in PBR [20]. Further targeted analyses are warranted to quantify the contribution of multiple gestations to the long-term trends in both PBR and LBWR in the country.

In the present study, prematurity emerged as the key determinant of the LBWR in Greece, with approximately two-thirds of all LBW births attributable to preterm delivery, and more than 70% of LBW neonates born preterm in 2023. Furthermore, the alarming upward trend in the LBWR observed since 1991 was found to be entirely explained at the population level by the corresponding increase in the PBR. This essentially means that prematurity is the central issue that must be addressed to achieve overall improvements in perinatal outcomes. Moreover, our findings indicate that the rise in LBWR is driven by the increasing proportion of newborns who are both preterm and LBW. This subgroup is of particular public health concern, as it represents the most vulnerable segment of the neonatal population, underscoring the need for multiple targeted interventions aimed at both prevention and improved clinical management [21,22].

The present analysis has the strength of being based on official national data derived from birth certificates, encompassing more than four decades of information from over 4.5 million live births. This extensive dataset made it possible to calculate and examine the trends in PLBWR and TLBWR separately, highlighting the increase in the proportion of preterm LBW neonates, who represent the highest-risk group. The impact of preterm births on the LBWR was assessed both through the PAR (%) and by estimating the proportion of preterm LBW births among all LBW births. Finally, the contribution of the rising PBR to the increase in LBWR was quantified.

However, this study has certain limitations. Although birthweight measurement is straightforward and highly accurate, the precision of gestational age dating may be subject to considerable error, particularly when the date of the last menstrual period is unknown or when ultrasound assessment is performed late in pregnancy. Moreover, potential changes over time in gestational age ascertainment or ultrasound-based dating may have influenced the classification of preterm births during the 1980-2023 period. Furthermore, due to the lack of relevant data, this analysis did not account for several important epidemiological factors - most notably pregnancy multiplicity, as well as maternal age, parity, infant sex, and socioeconomic variables [23-25] - which could be addressed in future research. Of note, these parameters should be studied as a priority with respect to their impact on the evolution of the PBR, given that from 1991 to 2023 the LBWR declined within both preterm and term birth categories.

## Conclusions

This nationwide analysis demonstrates that preterm birth is the principal driver of Greece’s high LBWR, with the population risk of LBW births attributable to prematurity rising sharply over the past three decades. The sustained increase in LBWR during this period is entirely explained by the concurrent rise in the PBR, underscoring the urgent need for targeted, evidence-based strategies to reduce prematurity and improve perinatal outcomes.
